# Plant architecture genes affect seed composition and seed weight in soybeans grown in the Midsouth USA

**DOI:** 10.3389/fpls.2026.1785158

**Published:** 2026-03-09

**Authors:** Nacer Bellaloui, James R. Smith, Jeffery D. Ray, Neeraj Kumar, Chunda Feng, Abdelraheem Abdelraheem

**Affiliations:** USDA, Agriculture Research Service, Crop Genetics Research Unit, Stoneville, MS, United States

**Keywords:** plant architecture, seed nutrition, seed oil, seed protein, soybean, stem termination

## Abstract

The Early Soybean Production System (ESPS) in the Midsouth is characterized by high humidity, high heat, water deficit, and disease pressure. Therefore, optimizing soybean plant architecture adapted to the ESPS is essential for maximizing seed yield (SY) and seed composition (nutrition). The objective of this research was to investigate the effect of plant architecture, i.e., stem termination genes on seed composition/nutrition (seed protein, oil, fatty acids, and sugars), and seed size (100-seed weight). We used a set of four near-isogenic stem termination lines from maturity group (MG) V that were adapted to the ESPS in the Midsouth. The four germplasm near-isogenic lines (NIL) are USDA-ARS-GDS-880*Dt1* (indeterminate), USDA-ARS-GDS-880dt1-*t2* (tall determinate), and USDA-ARS-GDS-880*Dt2* (semi-determinate), and determinate-*dt1*, with differing plant architectures derived from different stem termination genes. A 3-year field experiment was conducted in 2022, 2023, and 2024 at Stoneville. The results showed that the semi-determinate (semi-det), DS49-142, and Osage had higher seed protein content than the determinate (det), indeterminate (indet), and tall-determinate (tall-det) isolines in 2022, 2023, and 2024. Oil content in the semi-det was also higher than both checks, and comparable with the other genotypes. Oleic acid was higher in the semi-det and indet genotypes than in the det in 2022 and 2023. Raffinose content was higher in the tall-det and det isolines than in the semi-det isoline in 2022 and 2023, while stachyose was higher in the tall-det and indet isolines than in the semi-det type in 2023 and 2024. The 100-seed weight was highest in the semi-det, tall-det, and DS49–142 in all three years. Across the three years, and among the all lines, semi-det had the highest protein (38.95%), oleic acid (21.90%), and 100-seed weight (15.17 g). To our knowledge, this is the first report comparing these four plant architecture types in a MG V background in the ESPS in the Midsouth. This research demonstrated that the semi-det stem termination type contained the highest seed protein, acceptable levels of oil, and larger seed size than the other types. These are potentially desirable traits for soybean producers and the seed industry.

## Introduction

Increasing soybean seed yield (SY) using conventional breeding is slow, mainly due to the continued reliance on the narrow genetic base derived from the original ancestor lines (Hyten et al., 2006). Selection of higher SY in soybean resulted in cultivars with lower seed protein and higher seed oil content due to the inverse relationship between yield and protein, and the positive relationship between SY and oil content (Hurburgh et al., 1990; Heatherly and Smith, 2004; Eskandari et al., 2013; Rincker et al., 2014; Boehm et al., 2019; Diers et al., 2023). This was reported for MG II, III, and IV cultivars released from 1923 to 2008 in the north central U.S. (Rincker et al., 2014). In those studies, protein concentration decreased, oil concentration increased, and yield increased (Rincker et al., 2014; Diers et al., 2023). In the southern U.S., it was reported that MG V, VI, and VII cultivars, released from 1928 to 2008, showed less consistency in seed protein and oil than those of the north central region (Boehm et al., 2019). For example, although significant yield increases were recorded in the three southern MGs, a significant change of protein was recorded only in the MG VIs, but no significant pattern was shown for the other MGs for protein or oil content (Boehm et al., 2019; Diers et al., 2023). The continuous decline or inconsistency of protein levels in US soybean seed is a concern (Sonka et al., 2004; Lusas, 2004; Diers et al., 2023), as there are high protein meal standards to meet (48% protein meal that must meet a minimum standard of 47.5% protein).

Most of the MG II, III, and IV soybean cultivars tested above have an indeterminate plant architecture, whereas most of the MG V, VI, and VII cultivars tested above have a determinate plant architecture. The semi-determinate and tall determinate crop architectures mostly remain untested in both northern and southern production systems. Therefore, further testing is required to determine the optimal plant architecture for SY (Hartung et al., 1981; Cooper, 1971a, Cooper, 1971b) and seed composition. This is because plant architecture determines plant growth habit, height, number of branches, pods, seeds, and maturity, all of which can impact SY and seed quality (Reinhardt and Kuhlemeier, 2002; Hill, 2010; Sarlikioti et al., 2011; Li et al., 2014; Yao et al., 2015; Zhang et al., 2015; Pan et al., 2017; Zhang et al., 2021; Ding et al., 2022). Bernard (1972) reported that apical stem termination is under the genetic control of two loci (*Dt1* and *Dt2*). Cultivars with indeterminate stem termination are *Dt1Dt1dt2dt2*, and have typically been grown in the northern U.S., but in recent years, are being grown in the Early Soybean Production System (ESPS) in the southern U.S. Cultivars with determinate stem termination are *dt1dt1dt2dt2* and have a non-tapering apical stem at maturity, and typically more lateral branches than the indeterminate. Determinate cultivars are grown mostly in the southern U.S. but have also been grown in northern areas (Hartung et al., 1981). The semi-determinate stem termination is *Dt1Dt1Dt2Dt2* and is characterized by an apical stem at maturity that is intermediate in length between the indeterminate and determinate types. Likewise, the tall determinate (*dt1-tdt1-t dt2tdt2*) apical stem is intermediate in length to the indeterminate and determinate types, while shorter in length than the semi-determinate type.

It is well documented that most indeterminate cultivars are grown in the northern U.S. and Canada, whereas determinate cultivars are grown in the southern U.S. Studies on stem termination were previously conducted on MG II and III soybean (these alleles were backcrossed into NIL of indeterminate cv. Clark (*Dt1Dt1 dt2dt2*) (Bernard, 1972; Foley et al., 1986; Bernard et al., 1991; Cober and Tanner, 1995). Bernard (1972) studied the intermediate stem termination phenotype in T117 from USDA Genetic Type Collection, which he named “semi-determinate”. Bernard found that the semi-determinate trait in T117 was controlled by a dominant gene (*Dt2*) at a different locus than *Dt1*, and found that, phenotypically, (*Dt1Dt1Dt2Dt2*) was similar to the heterozygote *Dt1dt1dt2dt2*. The *Dt1* locus is epistatic with the *Dt2* locus; that is, *Dt2* is only expressed as semi-determinate in the presence of *Dt1*. Otherwise, it is masked in the presence of *dt1dt1*. Kim et al. (2022) also reported two genes regulating apical stem growth in soybean, *Dt1* and *Dt2*. In the *Dt1* genetic background, Dt2 genotypes have semi-determinate phenotypes. However, the recessive allele dt2 produces the indeterminate phenotype in the presence of *Dt1*. In the presence of a dt1 genetic background, the phenotype will be determinate, regardless of the alleles at the *Dt2* locus, which is an epistatic effect of the recessive dt1 allele with the *Dt2* alleles. Thompson et al. (1997) identified the tall determinate phenotype as *dt1-tdt1-t*, whether in the presence of *Dt2* or *dt2*, again showing epistasis between the two loci.

Researchers have identified genes, QTLs, molecular markers, candidate genes, and transcripts involved in stem termination phenotypes using either biparental lines (using segregating populations derived from two parental lines having difference for the trait) or genome-wide association studies (GWAS) (Grant et al., 2010; Abdel-Haleem et al., 2011; Wu et al., 2013; Xavier et al., 2017; Wang et al., 2019; Prince et al., 2020; Virdi et al., 2023; Chen et al., 2021; Kim et al., 2022; Clark et al., 2022; Wang et al., 2022; Diers et al., 2023).

Although the ESPS resulted in high SY under irrigated and non-irrigated conditions in the Midsouth (Heatherly, 1999), it often resulted in poor seed quality (Wrather et al., 1996; Heatherly and Bowers, 1999; Smith et al., 2008; Mengistu et al., 2010; Bellaloui et al., 2017) and seed composition alterations (Bellaloui et al., 2017, Bellaloui et al., 2021), due to high temperature, high humidity, and diseases, including *Phomopsis* (Smith et al., 2008). The conditions of high heat, high humidity, and Phomopsis seed decay result in seed damage, negatively impact on seed composition, and reduce seed quality, thereby increasing grain dockage at local grain elevators and reducing the economic return of farmers and producers. In addition, planting date showed to alter seed composition, especially in seed protein, oil, and leaf minerals content in ESPS, or had minimal effects (Morris et al., 2021; Miller et al., 2025). Therefore, it is critical that soybeans with high yield potential and high seed quality constituents be developed for the ESPS. This may be possible through the use of value-added genetic tolerance to seed deterioration but also needs to include the investigation of which crop architecture is optimal for each planting date and latitude environment. Identifying and utilizing the optimal combinations of crop architecture and planting date/production system will result in an increase in net returns to both U.S. soybean growers and U.S. grain/meal/oil processors/exporters.

Based on the above research, it is clear that plant architecture is genetically controlled and can significantly affect crop production (Agudamu et al., 2016; Burgess et al., 2019; Clark et al., 2022; Ding et al., 2022). Plant architecture determines branching pattern, size, shape, and number of leaves and flower organs, and internode distance (Reinhardt and Kuhlemeier, 2002). The comparison of the above four stem termination types in a mid MG V genotypic background has not previously been investigated in the ESPS of the midsouth, although recently, “semi-determinate” cultivars for MGs IV and V were developed and released for production in the Midsouth (Chen et al., 2022a, Chen et al., 2022b). However, the gene controlling the “semi-determinate” trait in Chen’s cultivars has not been disclosed. Chen et al. (2022a) developed and released a soybean cultivar ‘S16-11651C’ (Reg. no. CV-549, PI 699631), an early maturity group (MG) V, semi-determinate, conventional, high-yielding. This cultivar was tested in 94 environments in Missouri and 11 other mid-southern states from 2017 to 2020. The results showed that on average, S16-11651C yielded 4,327 kg/ha, which is 292 kg/ha higher than all tests mean of 4,035 kg/ha. Protein content of S16-11651C was 41.3% and oil was 22% on a dry weight basis. The cultivar S16-11651C has a weight of 13.5 g/100 seed. Chen et al. (2022a) indicated that S16-11651C has a high yield potential, high seed protein and oil content, and a broad adaptation across mid-southern states. In addition, Chen et al. (2022b) developed and released S16‐5540GT’ (Reg. no. CV‐551, PI 699633), a mid‐IV maturity group, semi‐determinate, glyphosate‐tolerant, high‐yielding soybean cultivar. The cultivar S16‐5540GT was tested in 88 environments in Missouri and other southern states from 2017 to 2020, and results showed that, on average, S16‐5540GT yielded 4,244 kg/ha, higher than all tests mean of 3,885 kg/ha. S16‐5540GT had an average of 41.1% seed protein and 22% oil on a dry seed weight basis. They indicated that S16‐5540GT is a pretty good choice for farmers who are interested in glyphosate tolerance for weed control and saving seed for planting.

**Figure 1:**
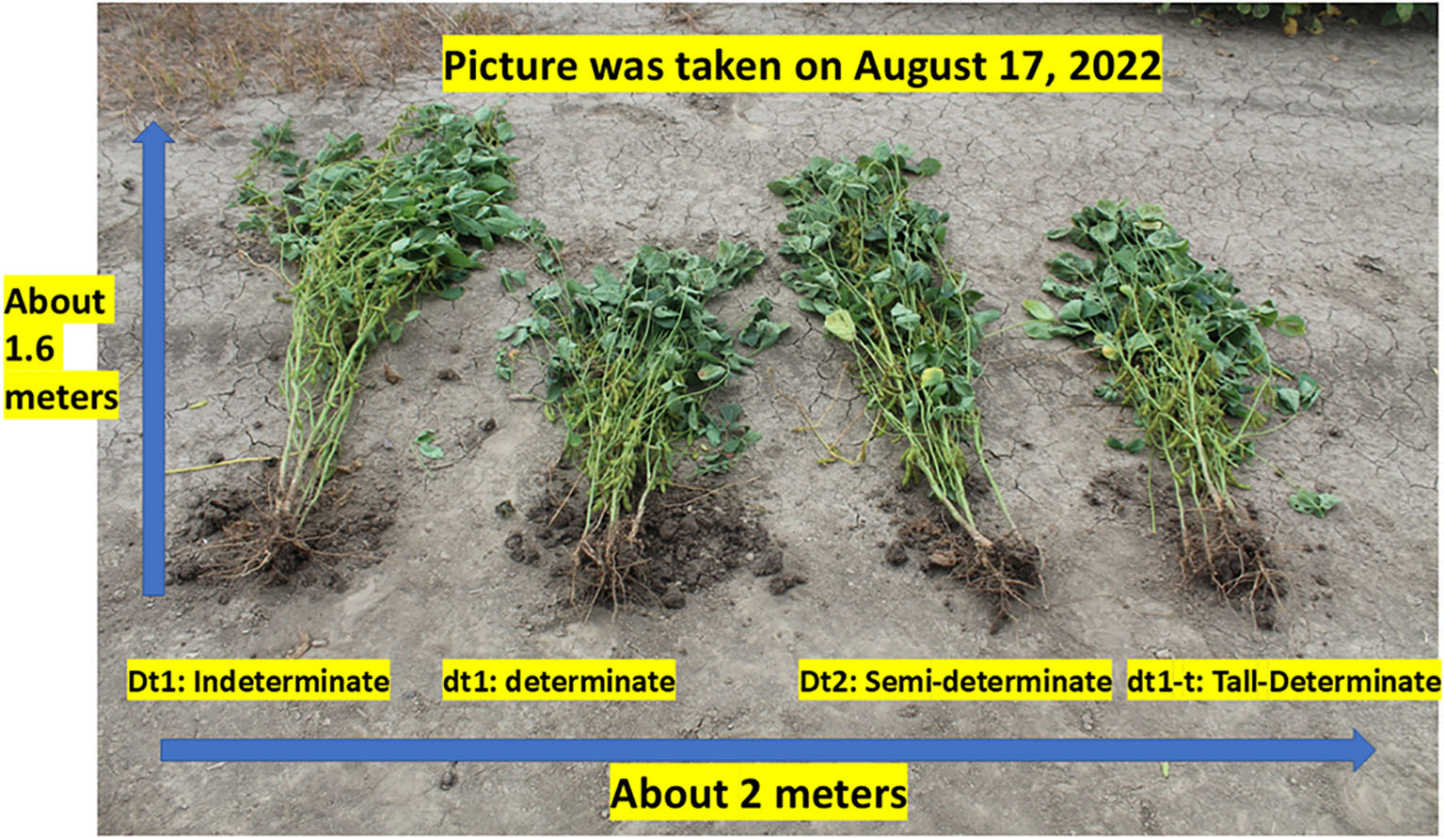
The four near-isolines of soybean maturity group V showing the different architectural growth types.

In spite of the importance of the stem termination genes on yield potential and seed quality, there is a lack of information on the effects of these genes on the seed composition and seed size. Therefore, our current research will investigate the effects of stem termination genes on seed composition and seed size as estimated by 100-seed weight in indeterminate (*Dt1, dt2*); semi-determinate (*Dt1, Dt2*); tall-determinate (dt1-t, dt2); determinate (*dt1, dt2*) soybeans of MG V. A set of mid V NIL in a DS-880 background was developed, where each line in the set differs in plant architecture/stem termination type, as listed above. Although previous research documented research on stem termination genes (determinate, indeterminate, semi-terminate, and tall determinate in multiple environments (Thompson et al., 1997; Heatherly, 1999; Wrather et al., 2003; Chen et al., 2022a, Chen et al., 2022b), to our knowledge, a set of mid MG V isolines was not previously available for study. These NIL (**Figure 1**) are adapted to the stress conditions of high heat, water deficient, and disease pressure of the midsouth growing conditions. The lines were released by the USDA-ARS in 2025, after which seed was entered into the USDA collection (Smith et al., 2025). The selection of these lines for this study was based on prior field experiments in Stoneville, MS, that showed that both the semi-determinate and tall-determinate had high yield potential in early plantings, with less seed damage and seed decay (data not shown).

## Materials and methods

### Breeding lines and growth conditions

The four used soybean NIL of MG V (mid MG V) are

USDA-ARS-GDS-880*Dt1* (indeterminate), USDA-ARS-GDS-880*dt1-t2* (tall determinate), and USDA-ARS-GDS-880*Dt2* (semi-determinate), and determinate (DS-880) (*dt1, dt2*). This set of isolines with differing plant architectures derived from different stem termination genes, was derived through backcrossing from determinate DS-880 (PI 659348) (https://npgsweb.ars-grin.gov/gringlobal/accessiondetail?id=1836762), where DS-880 was used as recurrent parent (RP) (Smith et al., 2010). The RP was previously released by ARS as an improved high yielding MG V germplasm with resistance to multiple diseases and adapted to the ESPS. To produce homogeneous BC4F5 for each plant architecture, five generations of self-fertilization and selection at Stoneville were conducted, and F5 were created in 2021. Each backcrossed line in the set has approximately 97% of the genotype of RP (DS-880). Therefore, the set essentially consists of four isolines that differ only in the combinations of the two stem termination genes (*Dt1*, *dt1*, or *dt1-t2*, and *Dt2 or dt2*) they carry. In addition to the two stem termination genes (Thompson et al., 1997) the four lines also likely have the same *E1* maturity allele (Langewisch et al., 2017), whose later maturity ensures time for more stem growth, more nodes with pods, and higher SY (Hartung et al., 1981). A three-year field experiment was conducted in 2022, 2023, and 2024 at the Delta Research and Extension Center at Stoneville, MS (W90” 53, N33” 25’). The experiment included the four isolines and two checks (DS49–142 and Osage). The experiment was furrow-irrigated, with a row spacing of 0.91m and a row length of 5.79m long planted. Planting dates were April 27 for 2022, April 13 for 2023, and April 16 for 2024. For 2023 and 2024, a three-row plot was used, but only the middle plot was sampled and harvested for seed analysis and for 100-seed weight. For 2022, the two border rows of the 4 row-plots were used, and rows were 5.79 m long at planting that were end-trimmed to 4.88 m between R5 and R6, with row-spacing of 0.91 m wide. 100-seed weight was used to estimate seed size.

### Seed composition analysis

Briefly, fully mature seeds were ground using a Laboratory Mill 3600 (Perten, Springfield, IL). Soybean seed protein, oil, fatty acids, and sugars were determined by a near-infrared reflectance spectrometer (NIRS™) DS3-F Analyzer using ISIscan™ Nova operating software (FOSS North America, Inc., Eden Prairie, MN, USA). Detectors used were Silicon (850–1100 nm) and Lead Sulfide (1100–2500 nm). The wavelength ranged from 850 to 2500 nm, and the number of subsamples was 7. Seed protein, oil, fatty acids, and sugars (sucrose, raffinose, stachyose) were expressed as a % and on a dry-weight basis.

### Experimental design and statistical analysis

The experiment was a randomized complete block design with three replicates. Each replicate (experimental unit) included all the stem termination lines and the two checks (genotypes). Analyses of variance (ANOVA) of year, line (genotype), and their interactions were conducted in SAS using PROC MIXED. Both year and genotype were considered as fixed effects, and replicates within years were considered as random effects (SAS, Statistical Analysis Systems, Cary, NC, USA, 2002–2012). Means were separated using Fisher’s protected Least Significant Difference (LSD) at 0.05. Correlation (R and P values) was conducted using PROC CORR in SAS, and the significant level was at 0.05.

## Results and discussion

Analysis of variance (ANOVA) showed that year had significant effect on oleic, linoleic, linolenic acids, raffinose, and stachyose (**Table 1**). Genotype (4 stem termination isolines and 2 checks) were significant for all seed composition components, except for raffinose, where it was significant for year, but not for genotype (**Table 1**). Year x genotype interactions were significant for protein, oleic and linolenic acids, sucrose, and raffinose, but not for oil and stachyose (**Table 1**). As years were different for all genotypes, and as the interactions were significant for most traits, the data will be presented and discussed by individual year.

**Table 1:** Analysis of variance (ANOVA)^a^ (F value and level of significance) for the effects of year, genotype, and their interactions for seed protein, oil, 100-seed weight(g), oleic acid, linoleic acid, linolenic acid, sucrose, raffinose, and stachyose (%) over the six genotypes (four isolines and two checks) evaluated over three years (2022, 2023, and 2024) in Stoneville, MS, USA.

Effect	DF	Protein (%)	Oil (%)	100-seed weight (g)	Oleic (%)	
		F value and level of significance				
Year (3)	2	7 ns	3.9ns	4.3ns	43.2***	
Genotype (6)	5	76.56***	85.8***	39.4***	8.1***	
Year*Genotype	10	4.71***	1.4ns	5.5***	5.1***	
Residuals	36					
Effect	DF	Linoleic (%)	Linolenic (%)	Sucrose (%)	Raffinose (%)	Stachyose (%)
Year (3)	2	23.0***	24.3***	2.8ns	11.3***	42.8***
Genotype (6)	5	22.5***	6.9***	36.1***	2.3ns	6.3***
Year*Genotype	10	0.8ns	5.4***	5.5***	2.8**	0.94
Residuals	36					

a *P ≤ 0.05; **P ≤ 0.01; ***P ≤ 0.001; ns, not significant.

Mean value of each genotype in 2022 showed that protein was higher in the semi-det plant type (38.2%) and in the checks than in RP (DS-880, 36.2%), and in the tall determinate (36.8%) and indeterminate (36.9%) plant types (**Table 2**). The Tall-det was not significantly different than the determinate or the indeterminate plant types. The reciprocal trend was noticed for oil content, as protein and oil have an inverse relationship (**Table 2**). Hundred-seed weight was greatest in the semi-det (16.7 g/100 seed) and tall-det (14.7 g/100 seeds) plant types, which were also different from each other, compared with the indeterminate (13.1 g/100 seed) and determinate (11.8 g/100 seed) plant types, which were also different from each other. Large differences in seed size appeared to be due to small changes at only two loci. Oleic acid content was higher in both the semi-det (21.2%) and indeterminate (21.2%) plant types than in the checks, tall-det (20.0%) and determinate (20.4%) plant types (**Table 3**). Generally, the opposite trend was noticed for linolenic acid, where linolenic acid was highest in the tall-det (8.9%) and determinate (8.7%) plant types. DS49–142 and the det plant type had the highest content of sucrose (both 5.7%) compared with the rest of the genotypes, with the semi-det (4.8) and Osage (4.9%) having the lowest levels of sucrose (**Table 4**). For raffinose content, the tall-det plant type was the highest (1.5%) and the semi-det plant type was the lowest (1.1%), with the other genotypes having intermediate values. Stachyose was highest in Osage (4.2%) and lowest in the tall determinate plant type (3.1%) (**Table 4**). The det (3.5%) and semi-determinate (3.4%) plant types had significantly more stachyose than the tall determinate plant type, but these differences may not be biologically meaningful.

**Table 2:** Mean values of each genotype for seed protein, oil, and 100-seed weight in four near-isogenic lines of maturity group (MG) V for stem termination types [Indeterminate (Indet); determinate (Det); tall-determinate (Tall-Det); semi-determinate (Semi-Det)]; and check1(DS49-142); check2 (Osage).

Year/Genotype	Protein (%)	Oil (%)	100-seed weight (g)
2022			
DS49-142	41.7	19.2	14.2
Osage	41.0	19.45	13.2
Indeterminate	36.9	22.5	13.1
Determinate	36.2	22.3	11.8
Semi-determinate	38.2	21.8	16.7
Tall-determinate	36.8	21.8	14.7
LSD	0.598	0.207	0.421
2023			
DS49-142	43.5	20.6	13.0
Osage	41.6	20.8	9.9
Indeterminate	40.1	23.1	12.7
Determinate	38.3	22.5	11.1
Semi-determinate	40.2	22.5	13.0
Tall-determinate	38.1	22.3	11.8
LSD	0.294	0.256	0.396
2024			
DS49-142	40.0	20.5	14.4
Osage	40.6	20.7	11.5
Indeterminate	33.8	23.4	12.1
Determinate	35.9	23.0	12.6
Semi-determinate	38.2	22.7	16.3
Tall-determinate	35.5	22.6	14.7
LSD	0.422	0.225	0.370

LSD, Least significant difference; significant level was at 5%. The four used soybean near-isogenic lines of MG V (mid MG V) are: USDA-ARS-GDS-880Dt1 (indeterminate), USDA-ARS-GDS-880dt1-t2 (tall determinate), and USDA-ARS-GDS-880Dt2 (semi-determinate), and determinate (DS-880) (dt1, dt2). This set of isolines with differing plant architectures derived from different stem termination genes, was derived through backcrossing from determinate DS-880 (PI 659348). The experiment was conducted in 2022, 2023, 2024 in Stoneville, MS, USA.

**Table 3:** Mean values of each genotype for seed fatty acids (oleic, linoleic, and linolenic) in four near-isogenic lines of maturity group (MG) V for stem termination types [Indeterminate (Indet); determinate (Det); tall-determinate (Tall-Det); semi-determinate (Semi-Det)]; and check1(DS49-142); check2 (Osage).

Year/Genotype	Oleic (%)	Linoleic (%)	Linolenic (%)
2022			
DS49-142	20.3	49.3	7.8
Osage	20.4	48.4	7.9
Indeterminate	21.2	47.4	7.8
Determinate	20.4	47.2	8.7
Semi-determinate	21.2	47.9	8.2
Tall-determinate	20.0	47.6	8.9
LSD	0.380	0.215	0.320
2023			
DS49-142	22.7	49.7	8.4
Osage	21.7	49.5	9.3
Indeterminate	23.7	48.2	7.4
Determinate	20.9	48.0	10.1
Semi-determinate	22.4	48.8	9.0
Tall-determinate	21.3	48.4	9.6
LSD	0.259	0.209	0.275
2024			
DS49-142	21.3	48.5	8.9
Osage	21.4	48.5	9.0
Indeterminate	20.7	47.2	10.2
Determinate	21.3	47.1	9.2
Semi-determinate	21.9	48.2	9.2
Tall-determinate	21.1	47.9	9.5
LSD	0.192	0.290	0.230

LSD, Least significant difference; significant level was at 5%. The four used soybean near-isogenic lines of MG V (mid MG V) are: USDA-ARS-GDS-880*Dt1* (indeterminate), USDA-ARS-GDS-880*dt1-t2* (tall determinate), and USDA-ARS-GDS-880Dt2 (semi-determinate), and determinate (DS-880) (*dt1, dt2*). This set of isolines with differing plant architectures derived from different stem termination genes, was derived through backcrossing from determinate DS-880 (PI 659348). The experiment was conducted in 2022, 2023, 2024 in Stoneville, MS, USA. .

**Table 4:** Mean values of each genotype for seed sucrose, raffinose, and stachyose in four near-isogenic lines of maturity group (MG) V for stem termination types [Indeterminate (Indet); determinate (Det); tall-determinate (Tall-Det); semi-determinate (Semi-Det)]; and check1(DS49-142); check2 (Osage).

Year/Genotype	Sucrose (%)	Raffinose (%)	Stachyose (%)
2022			
DS49-142	5.67	1.36	3.38
Osage	4.93	1.38	4.18
Indeterminate	5.52	1.39	3.28
Determinate	5.66	1.22	3.45
Semi-determinate	4.84	1.05	3.41
Tall-determinate	5.46	1.49	3.08
LSD	0.108	0.260	0.220
2023			
DS49-142	5.43	0.50	4.28
Osage	5.18	0.89	4.76
Indeterminate	5.06	0.60	4.48
Determinate	5.91	1.19	4.11
Semi-determinate	5.16	0.72	4.23
Tall-determinate	5.76	0.79	4.41
LSD	0.065	0.183	0.151
2024			
DS49-142	5.68	0.70	3.85
Osage	5.12	1.32	4.37
Indeterminate	5.36	2.01	4.04
Determinate	5.44	0.81	4.00
Semi-determinate	4.76	1.04	3.85
Tall-determinate	5.45	0.97	3.86
LSD	0.082	0.135	0.128

LSD, Least significant difference; significant level was at 5%. The four used soybean near-isogenic lines of MG V (mid MG V) are: USDA-ARS-GDS-880Dt1 (indeterminate), USDA-ARS-GDS-880*dt1-t2* (tall determinate), and USDA-ARS-GDS-880*Dt2* (semi-determinate), and determinate (DS-880) (*dt1, dt2*). This set of isolines with differing plant architectures derived from different stem termination genes, was derived through backcrossing from determinate DS-880 (PI 659348). The experiment was conducted in 2022, 2023, 2024 in Stoneville, MS, USA.

In 2023, similar observations for protein content were observed in that protein was higher in the semi-det type (40.2%) and checks than in the det (38.3%) and tall determinate (38.1%) types, and oil was higher in the semi-det (22.5%), det (22.5%), indeterminate (23.1%), and tall-det (22.3%) types than in the checks (**Table 2**). For 100-seed weight, the semi-determinate (13.0 g/100 seed) and indeterminate (12.7 g/100 seed) had larger seeds than the tall-det and determinate types (11.8 and 11.1 g/100 seed) (**Table 2**). Oleic acid was higher in the semi-det (22.4%), indet (23.7%), and DS49-142 (22.7%) than in Osage (21.7%), the tall-det (21.3%), and the det (20.9%) (**Table 3**). Levels of linolenic acid were correspondingly reciprocal to those of oleic acid, with the determinate type at 10% and the indeterminate at 7.4%. Seed sucrose and raffinose contents were higher in the determinate type (5.9 and 1.2%, respectively) than in the indeterminate type (5.1 and 0.6%, respectively), with the tall determinate type of intermediate (5.8 and 0.8%, respectively) and the semi-determinate type similar (5.2 and 0.7%, respectively) to the indeterminate (**Table 4**). Stachyose levels were inversely related to sucrose and raffinose levels in the indeterminate (4.5%) and determinate (4.1%) types, but not in the tall-det (4.4%) and semi-det (4.2%) types (**Table 4**).

In 2024, among the four plant architectures, protein was highest, and oil was lowest in the semi-determinate type (38.2 and 22.2%, respectively), whereas the indeterminate type had the lowest protein and the highest oil (33.8 and 23.4%, respectively). The tall determinate (35.5 and 22.6%, respectively) and the determinate (35.9 and 23.0%, respectively) types were intermediate (**Table 2**). The 100-seed weight was higher in the semi-det (16.3 g/100 seed) than in the tall-det (14.7 g/100 seed), determinate (12.6 g/100 seed), and indeterminate (12.1 g/100 seed) types. DS49–142 was intermediate for seed size (14.4 g/100 seed), whereas Osage had the smallest seed (11.5 g/100 seed) (**Table 2**). Again, among the four types, oleic acid was the highest and linolenic acid the lowest in the semi-det type (21.9 and 9.2%, respectively), whereas for the indeterminate type, the opposite was true (20.7% oleic acid and 10.2% linolenic acid). The determinate type (21.3 and 9.2%, respectively) and the tall determinate type (21.1 and 9.5%, respectively) were again intermediate (**Table 3**). In 2024, sucrose was higher in the tall-det and determinate types (5.5 and 5.4%, respectively), and lowest in the semi-det type (4.8%). DS49–142 had the highest sucrose (5.7%), whereas the indeterminate and Osage (5.4 and 5.1%, respectively) were intermediate (**Table 4**). Among the four architecture types, the indeterminate type had the highest raffinose and stachyose (2.0 and 4.0%, respectively), which were significantly higher than those of the tall determinate (1.0 and 3.9%, respectively) and semi-determinate (1.0 and 3.9%, respectively) types. The determinate had the lowest raffinose (0.8%) but was not different than the indeterminate for stachyose (4.0%). (**Table 4**).

### Correlation between seed composition components

In all three years, there was a negative correlation between protein and oil (*P* ≤0.001) (**Table 5**), oleic acid and linolenic acid (*P* ≤ 0.001), and between oleic acid and raffinose (*P* ≤0.05) (**Table 5**). There was also a negative correlation between raffinose and stachyose (*P* ≤0.05) in 2022 and 2023. There was a positive association between sucrose and raffinose in 2022 and 2023, but not in 2024 (**Table 5**). However, there was a positive correlation between linolenic acid and raffinose (*P* ≤0.01) (**Table 5**). Seed size was not associated with any seed composition trait in 2022 and 2024, except that it was negatively associated with stachyose in 2023 and 2024. In 2023, seed size was also negatively associated (*P* ≤0.05) with raffinose, sucrose, and linolenic acid, while being positively associated (*P* ≤0.05) with oleic acid.

**Table 5:** Correlation (R and P values)^a^ between seed composition components (%) and 100-seed weight (g/100 seed) of six soybean genotypes in three years.

2022							
Variable	Protein	Oil	Oleic	Linolenic	Sucrose	Raffinose	Stachyose
Oil	-0.94***						
Oleic	ns	ns					
Linolenic	ns	ns	-0.72***				
Sucrose	ns	ns	ns	ns			
Raffinose	ns	ns	-0.79***	0.57*	ns		
Stachyose	ns	ns	ns	-0.65**	-0.64**	-0.59*	
100-seed weight	ns	ns	ns	ns	ns	ns	ns
2023							
	Protein	Oil	Oleic	Linolenic	Sucrose	Raffinose	Stachyose
Oil	0.73**						
Oleic	ns	ns					
Linolenic	ns	ns	-0.97***				
Sucrose	-0.47*	ns	-0.78***	0.70***			
Raffinose	ns	ns	ns	ns	0.49*		
Stachyose	ns	ns	0.54*	-0.42*		-0.55*	
100-seed weight	ns				ns	ns	-0.43*
2024							
	Protein	Oil	Oleic	Linolenic	Sucrose	Raffinose	Stachyose
Oil	-0.94***						
Oleic	0.50*	ns					
Linolenic	-0.66***	0.60**	-0.83**				
Sucrose	ns	ns	-0.61*	ns			
Raffinose	ns	ns	-0.61*	0.80***	ns		
Stachyose	ns	ns	ns	ns	ns	ns	
100-seed weight	ns	ns	ns	ns	ns	ns	-0.50*

a *P ≤ 0.05; **P ≤ 0.01; ***P ≤ 0.001.

**Figure 2:**
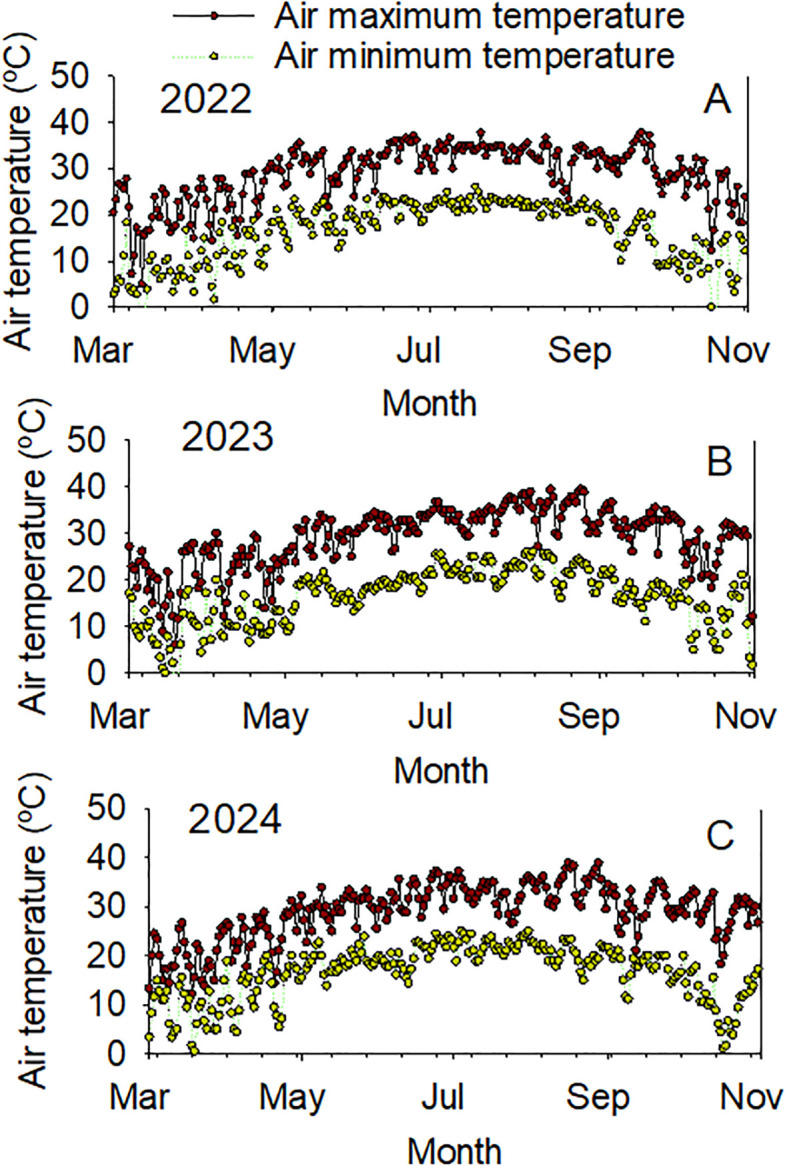
Daily weather components (maximum air temperature (°C), Max Temp; minimum air temp (°C), Min Temp in years 2022 (top), 2023 (middle), 2024 (bottom).

**Figure 3:**
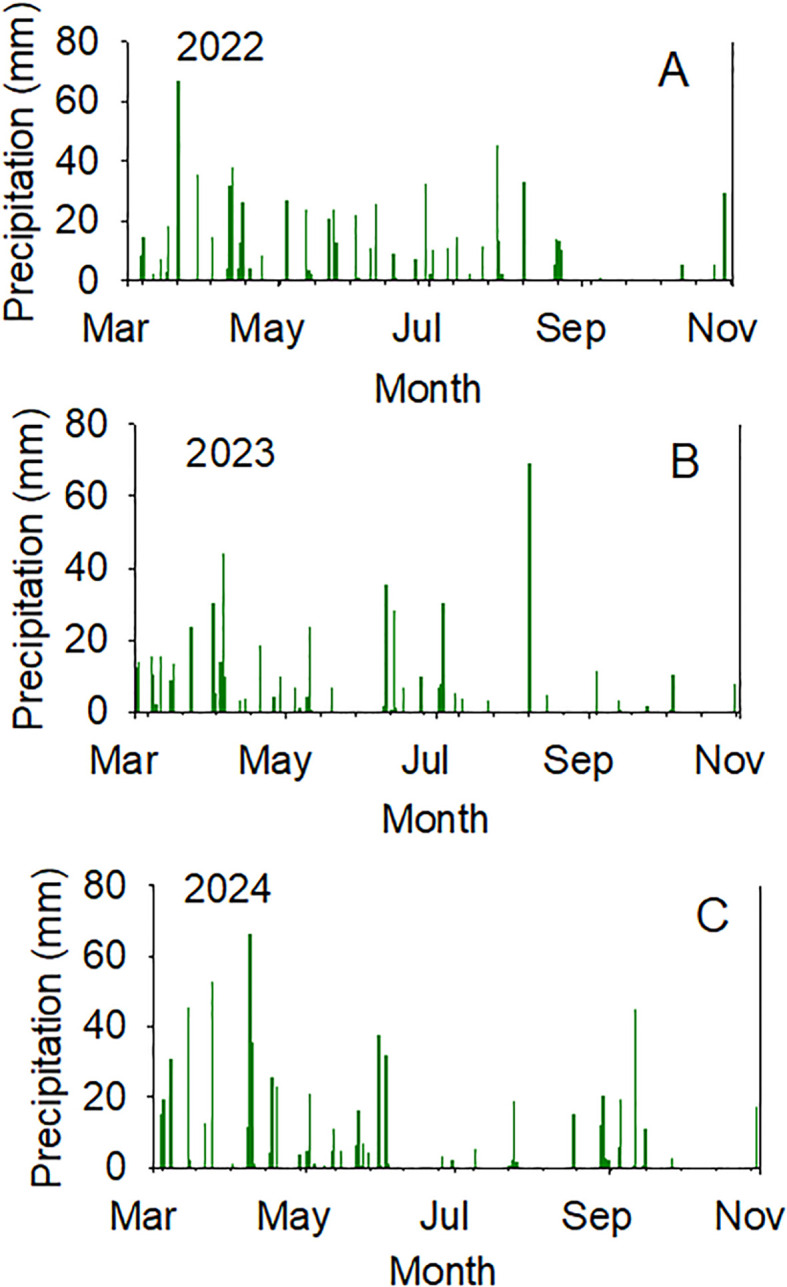
Daily weather components (precipitation (mm) in years 2022 (top), 2023 (middle), 2024 (bottom).

The significant effects of year and genotype for the seed composition components indicated the importance of these factors for the contents of protein, oil, fatty acids, and sugars in seeds, as well as seed size. The significant interactions of year-by-genotype for some components indicated that the changes in planting date and weather components in each year, especially rainfall and temperature, could be major sources of these changes in these components (**Figures 2**, **3**). The pattern and distribution of temperature and rainfall were different in each year. For example, in 2022 in July, it was 34.1°C air maximum temperature (maxT), 22.8°C air minimum temperature (minT), and rainfall (3.6 mm); in August, however, 32.1°C air maximum temperature (maxT), 21.8°C air minimum temperature (minT), and rainfall (2.5mm). In 2024 in July, it was 32.6°C air maximum temperature (maxT), 22.2°C air minimum temperature (minT), and rainfall (1.0 mm); in August, however, 34.5°C air maximum temperature (maxT), 20.5°C air minimum temperature (minT), and rainfall (1.7 mm). It was reported that environmental conditions can modify protein and oil concentrations (Assefa et al., 2019) by about 20% (Rotundo and Westgate, 2009).

The higher seed protein content in the semi-det than in the det, indet, and tall-det types in 2022, 2023, and 2024 showed the consistency of the semi-det version in accumulating higher protein in the seed compared with the other genotypes. The semi-determinate was also more competitive than the other plant architectures, with the high-protein check DS49–142 and with Osage. Oil content in the semi-det was higher than both checks, and competitive with the rest of the genotypes, reflecting the higher stability of seed protein and oil content in these genotypes compared with the others. The higher content of oleic acid in the semi-det and indet genotypes than in the det for two years (2022 and 2023), and the lower linolenic acid in the semi-det compared to the det in 2022 and 2023, indicated the inverse relationship between oleic and linolenic acid. Also, the higher oleic acid in the semi-det may reflect a heat tolerance response to higher temperature (Narayanan et al., 2020). Higher oleic acid with lower linolenic acid is a desirable trait in soybean seed for the seed industry as oleic acid contributes to oil stability and longer shelf-life (Pham et al., 2012). Sucrose, raffinose, and stachyose were higher in the tall-det and det types compared with the semi-det type. Sucrose contributes to flavor and taste, and higher raffinose and stachyose are involved in desiccation/dehydration and in tolerance to stress conditions such as cold, drought, high heat, and diseases (Ren et al., 2009; Obendorf, 1997). The higher stachyose content in tall-det and det types and lower in semi-det may suggest an inverse relationship between stachyose and seed-weight; however, further research is needed before a conclusive conclusion is made as a negative relationship between stachyose and seed-weight was shown only in two years out of three years. The consistently higher 100-seed-weight in the semi-det and tall-det types compared with the other stem termination genotypes, including checks, indicates a potentially large pleiotropic effect from changes at just two loci.

Information on the effect of stem termination genes on seed composition and seed size is insufficient. What is available in the literature is information related to the effect of node position or type of branch on seed protein, oil, and sugars (Bennett et al., 2003; Guleria et al., 2007, Guleria et al., 2008; Bellaloui and Gillen, 2010). For example, it was found that seeds developed in the upper one-fourth of the main stem of the plant had a higher concentration of proteins and a lower concentration of oil than from the lower one-fourth of the main stem of the plant (Collins and Cartter, 1956; Escalante and Wilcox, 1993a, Escalente and Wilcox, 1993b). This was explained to be due to environmental factors (Wolf et al., 1982; Maestri et al., 1998) and nutrient translocation supply (Nakasathein et al., 2000; Bennett et al., 2003). Rosso et al. (2021), using four genotypes with contrasting branching (P31T11R, MG 3.1, released in 2014; P34T43R2 (MG 3.4, 2014); P35T58R (MG 3.5, 2013); and P39T67R (MG 3.9, 2013) (Corteva Agriscience, Johnston, Iowa, US), reported that information on seed composition at different canopy locations such as main stem and branch nodes is lacking, and consequently they investigated the effects of SY and composition (protein, oil, fatty acids, and amino acids) at the main stem and stem branches in four genotypes at three main stem segments (lower, middle, and upper). They found that the upper main stem had higher protein (25%) and oil (15%) content compared to the lower portion. They further indicated that oil concentration increased from top to bottom, while protein concentration followed the opposite trend. Amino acids lysine, cysteine, methionine, threonine, and tryptophan were higher in the lower main stem, while the oleic/(linoleic + linolenic) ratio was higher in the upper portions, and branches produced seeds with lower nutritional quality than the main stem. Also, the contribution of branches to yield (%) was positively related to limiting amino acid abundance and oil concentration across soybean genotypes. Upper main stem nodes had higher protein and lower oil concentration than the lower main stem in another study (Sharma et al., 2013). However, little is known about the concentration of seed components in the branches (Rosso et al., 2021). Rosso et al. (2021) and Escalante and Wilcox (1993a, EscalanteWilcox, 1993b) reported that protein and oil vertical canopy profiles are consistent regardless of determinate or indeterminate plant architectures and overall genotype protein level (Escalante and Wilcox, 1993a, EscalanteWilcox, 1993b). A higher concentration of sulfur-containing amino acids was observed in the seeds of the lower main stem, while oleic acid was higher in seeds of the upper segment of the main stem (Bennett et al., 2003; Bellaloui and Gillen, 2010). It has been reported that continuous SY increases have resulted in decreases in protein content and increases in seed oil content (Rincker et al., 2014), which could be due to the complex interactions between genetics and the environment for stem branching in soybeans (Shim et al., 2017, Shim et al., 2019). It was also suggested that differences in the source for C assimilation could influence seed composition of specific stem segments, mainly for protein, due to the nitrogen remobilization process (Sinclair and De Wit, 1975). Our research showed that both the semi-determinate and the tall determinate types are strong candidates for protein and oil accumulation, in that the semi-det had higher protein and was still competitive for oil and also does not compromise the level of sugars, as its level of sugars was within the normal level of currently used cultivars. Since the sugars levels in soybean seed is relatively wide, it is difficult to conclude that the existing levels of sugars in the current genotypes are low or high. However, it can be safely report that these amounts play their physiological and metabolic roles because they fall in the acceptable levels of soybean cultivars/genotypes. For example, the average soybean seed contains 4 to 5% sucrose, 1 to 2% raffinose, and 3.5 to 4.5% stachyose (Wilson, 1995). Previous studies on stem termination have not compared the four mid MG V stem termination isolines described in the current paper for seed composition and seed size. To our knowledge, these four lines are the first set of released NIL for stem termination genes (Dt1, dt1, dt1-t2, and Dt2) in a mid V background. Therefore, we believe this research contributes new knowledge and important information to farmers and seed processors.

## Conclusions

This study demonstrates that stem termination type can play a critical role in seed size and seed composition. In April-plantings over three years in Mississippi, the higher protein content and the largest seed size was observed in the semi-determinate type (**Tables 6**, **7**). The semi-determinate type, along with checks, also had the highest seed protein content in each year (**Table 2**). Although oil content in the semi-det was higher than both checks and competitive with the rest of genotypes, the indeterminate type had the highest oil in each year. As in other studies, protein and oil were found to be inversely proportional to each other in this study, as were also oleic acid and linolenic acid. Specific rankings of isolines varied across years for oleic and linolenic acids, likely due to variations in temperature patterns across years. Significant year interactions among the isolines for sucrose and stachyose also resulted in rank variations across years, again likely due to weather variations. Line rankings for raffinose varied across years, even though there were no significant year-by-line interactions. Employing a semi-determinate soybean in a MG V background in the ESPS may be an effective tool to help farmers increase their seed protein and seed size. Larger seed size may not be advantageous for reducing mature seed damage, but increased protein content would be beneficial to world markets. Further research is still needed to evaluate the effect of MG V stem termination phenotypes on SY in the ESPS.

**Table 6:** Mean values of the four near-isogenic lines for seed protein, oil, yield, fatty acids (oleic, linoleic, and linolenic), sugars (sucrose, raffinose, and stachyose) in four near-isogenic lines of maturity group (MG) V for stem termination types [Indeterminate (Indet); determinate (Det); tall-determinate (Tall-Det); semi-determinate (Semi-Det)] over three years.

Genotype	Protein (%)	Oil (%)	Yield (g)	Oleic (%)	Linoleic (%)	Linolenic (%)	Sucrose (%)	Raffinose (%)	Stachyose (%)
Indeterminate	37.0	23.0	12.6	21.9	47.6	8.49	5.31	1.33	3.93
Determinate	36.8	22.6	11.9	20.9	47.4	9.32	5.67	1.07	3.85
Semi-determinate	38.9	22.2	15.2	21.9	48.4	8.85	4.93	0.92	3.88
Tall-determinate	36.8	22.2	13.8	20.8	48.0	9.36	5.56	1.09	3.78
LSD	0.5	0.2	0.4	0.3	0.2	0.27	0.07	0.15	0.16

The experiment was conducted in 2022, 2023, 2024 in Stoneville, MS, USA.

**Table 7:** Ranking of seed protein, oil, yield, fatty acids (oleic, linoleic, and linolenic) and sugars (sucrose, raffinose, stachyose) in four near-isogenic lines of maturity group (MG) V for stem termination types [Indeterminate (Indet); determinate (Det); tall-determinate (Tall-Det); semi-determinate (Semi-Det)] across three years.

Genotype	Protein	Oil	Yield	Oleic	Linoleic	Linolenic	Sucrose	Raffinose	Stachyose
Indeterminate	2	1	3	2	3	4	3	1	1
Determinate	3	2	4	3	4	2	1	3	3
Semi-determinate	1	4	1	1	1	3	4	4	2
Tall-determinate	4	3	2	4	2	1	2	2	4

The experiment was conducted in 2022, 2023, 2024 in Stoneville, MS, USA.

## Data Availability

The datasets presented in this study can be found in online repositories. The names of the repository/repositories and accession number(s) can be found in the article/**Supplementary Material**.
