# Supplementary material for: Plant architecture genes affect seed composition and seed weight in soybeans grown in the Midsouth USA
Source: Front Plant Sci. 2026 Mar 9;17:1785158. doi: 10.3389/fpls.2026.1785158 (PMC13006637; doi:10.3389/fpls.2026.1785158)

Supplementary Figure S1: Mean values in 2022 for seed protein, oil, oleic and linolenic acids (A), seed sucrose, raffinose, and stachyose, and 100-seed weight (B) among the four near-isogenic lines (indeterminate=indeterm; determinate=determin; semi-determinate=semi-det; tall-determinate=tall-det), and check1= DS49-142 (high-germination germplasm) and check2= MG V cultivar Osage.

A

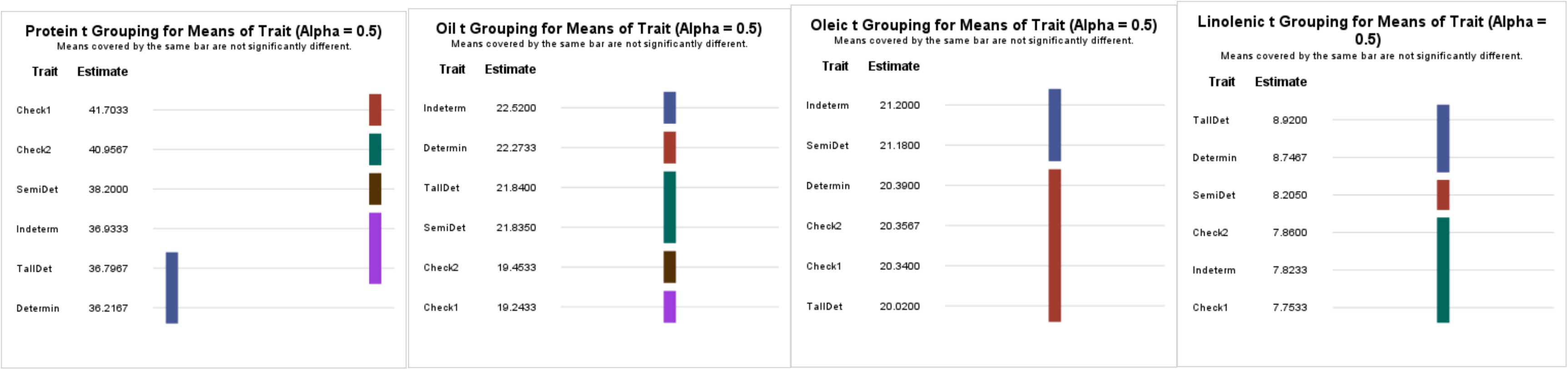

B

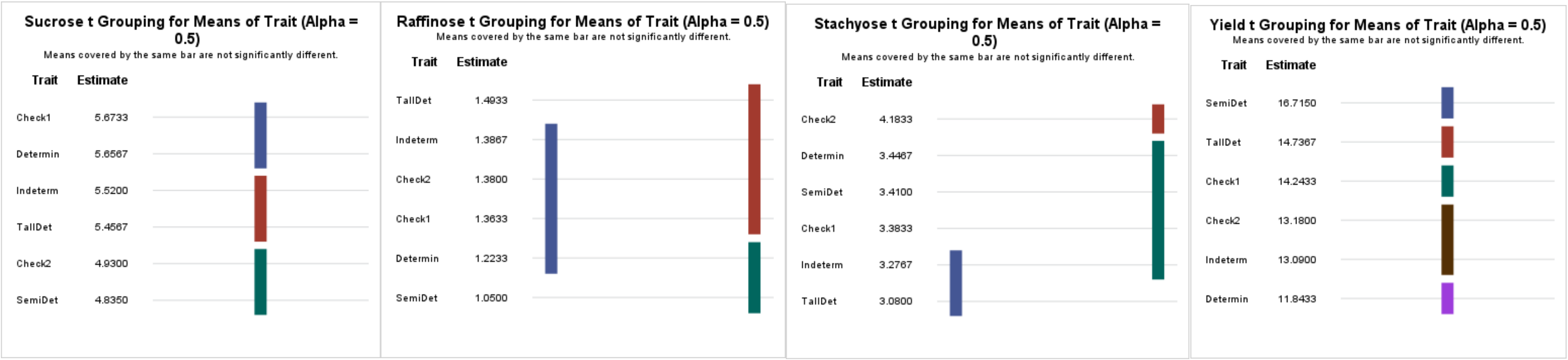

Supplement: Supplementary file 1 [file DataSheet1.pdf]
